# Relations between the school physical environment and school social capital with student physical activity levels

**DOI:** 10.1186/1471-2458-13-1191

**Published:** 2013-12-17

**Authors:** Brenton Button, Stephen Trites, Ian Janssen

**Affiliations:** 1 School of Kinesiology & Health Studies, Queen’s University, Kingston, K7L 3N6 Ontario, Canada; 2 Department of Public Health Sciences, Queen’s University, Kingston, K7L 3N6 Ontario, Canada

**Keywords:** Adolescent, Physical activity, School, Built environment, Social capital

## Abstract

**Background:**

The physical and social environments at schools are related to students’ moderate-to-vigorous physical activity (MVPA) levels. The purpose of this study was to explore the interactive effects of the school physical environment and school social capital on the MVPA of students while at school.

**Methods:**

Data from 18,875 grade 6–10 students from 331 schools who participated in the 2009/10 Canadian Health Behaviour in School-Aged Children survey were analyzed using multi-level regression. Students answered questions on the amount of time they spend in MVPA at school and on their school’s social capital. Administrator reports were used to create a physical activity related physical environment score.

**Results:**

The school physical environment score was positively associated with student MVPA at school (β = 0.040, *p* < .005). The association between the school social capital and MVPA was also positive (β = 0.074, *p* < .001). The difference in physical environments equated to about 20 minutes/week of MVPA for students attending schools with the lowest number of physical environment features and about 40 minutes/week for students attending schools with the lowest school social capital scores by comparison to students attending schools with the highest scores.

**Conclusions:**

The findings suggest that school social capital may be a more important factor in increasing students MVPA than the school physical environment. The results of this study may help inform interventions aimed at increasing student physical activity levels.

## Background

Surveillance data from 105 countries indicates that 80% of 13–15 year olds do not meet the public health guideline of 60 minutes of daily moderate-to-vigorous physical activity (MVPA) [1]. Increasing the rates of MVPA amongst young people is important because MVPA in this age group is linked to a decrease in chronic disease risk factors such as obesity, the metabolic syndrome, and high blood pressure [2]. Furthermore, MVPA improves academic performance, increases self-esteem, and decreases the likelihood of experiencing mental health problems [3,4].

Schools are an important setting for MVPA for youth because they spend large amounts of time there and because school-based physical activity opportunities are accessible to all students [5]. There are numerous opportunities for MVPA at school such as physical education classes and several non-curricular options (e.g., active play at recess, intramural sports, and varsity sports). While the course load is similar at most schools, the MVPA levels of students vary from school to school [5]. This difference could potentially be explained by differences in the physical environments at school and/or the level of school social capital.

There are several features of the school physical environment that are relevant for MVPA such as gymnasiums, sports fields, and fitness rooms. Previous research has shown that the school physical environment is associated with student MVPA levels, although such associations are modest in strength and not consistent in all population groups [6-8]. For instance, a cross-sectional study of 7,638 grade 6–10 students found girls attending high schools with 5 or 6 physical activity related physical environment features were 62% more likely to participate in MVPA than girls attending schools with one or no features; this difference was only 10% in boys [9].

In its most basic terms social capital refers to the social connections that people have [10] and it has been hypothesized that social capital impacts physical activity participation through enhanced communication [11]. If a school has enhanced communication it could be easier for students to find out about available physical activity opportunities. A number of studies have shown a positive albeit weak association between social capital and physical activity [11-14]. For example, a cross-sectional study of 680 American adolescents reported by comparison to adolescents living in neighbourhoods with a high social cohesion were only half as likely to be inactive than adolescents living in neighbourhoods with a low social cohesion [11].

Previous research based on the neighbourhood environment suggests that social capital may moderate the influence of the physical environment on physical activity. Specifically, a study of 12–13 year olds from the Netherlands found that each one standard deviation increase in a neighbourhood social capital score was associated with a 50% increased odds of leisure-time sports participation within neighbourhoods with lots of park space; this association was not observed in neighbourhoods with a limited amount of park space [12]. To our knowledge, previous research has not simultaneously considered the associations between the physical environment and social capital at school with student physical activity levels.

The purpose of this study was to examine the independent and interactive associations between the school physical environment and school social capital with the MVPA levels of students during the school day. These findings could help direct intervention efforts. We had the opportunity to examine these relationships in a large and representative sample of Canadian youth in grades 6–10.

## Methods

### Study setting, design, and participants

This research involved analyses of cross-sectional data from the 2009/10 Canadian Health Behaviour in School-Aged Children Survey (HBSC). The Canadian HBSC was conducted in collaboration with the World Health Organization and followed an established international protocol [15]. HBSC participants consisted of students in grades 6 to 10 (approximately 11–15 years old) in publicly funded schools across Canada. Youth attending private, special needs, or home schools were ineligible, as were institutionalized, incarcerated or homeless youth. The survey used a cluster sampling design, with classrooms reflecting the distributions of schools according to size, location, language, and religion [15]. The survey gathered information on 26,078 students from 436 schools. The General Research Ethics Board of Queen’s University granted ethics approval for the study. Individual schools, parents and guardians, and the student participants provided consent. Seventy seven percent of eligible students participated.

The main component of the HBSC was a student questionnaire that collected information on students’ demographics, health behaviours, health determinants, and health outcomes. In addition, the school principal or designate (e.g., vice principal or another person with an upper level administrative position) completed an administrator questionnaire, which inquired about the schools characteristics including the size and demographic distribution of the students, policies, programs, and availability of facilities. Information from the administrator survey was linked to the individual–level information obtained from the student participants.

For the present study, we excluded 4,737 students from 105 schools as the administrator questionnaire was either not completed or was missing data for one or more of the relevant study variables. An additional 2,466 students were excluded because of missing physical activity or covariate information. Thus, the final sample consisted of 18,875 students from 331 schools. Compared to the 7,203 students who were excluded, the final sample was similar in age (0.02 years younger), gender distribution (49.4% vs. 48.0% male), and socioeconomic status.

### Physical activity at school

The outcome of interest was participation in MVPA at school. The student questionnaire asked “*About how many hours a week do you usually take part in physical activity that makes you out of breath or warmer than usual in your class time at school?*” and “*About how many hours a week do you usually take part in physical activity that makes you out of breath or warmer than usual in your free time (for example, lunch) at school?*”. There were nine response options for each question that ranged from “*none at all*” through “*7 or more hours*”. Responses from the two questions were combined to create a continuous score that ranged from 0 to 14 hours/week. A panel of physical activity experts in the international HBSC assembly developed these physical activity questions based on face validity, with the intent that they be interpretable by 11–15 year olds from a variety of countries [15]. A previous validation study on physical activity questions similar to those used in the HBSC reported that questionnaire responses were modestly correlated with objective measures of physical activity obtained by accelerometry (r = 0.39) [16,17], although it is important to recognize that questionnaires and accelerometers measure different aspects of physical activity (e.g., questionnaires measure time spent doing an activity, including sedentary and light intensity time, while accelerometers measure all movement at a defined intensity, including bouts of activity and sporadic activity).

### School physical environment

The HBSC administrator survey asked if students had access to the following physical activity facilities on school grounds: (1) gymnasium, (2) other large room suitable for physical activity, (3) fitness room for aerobic or strength training, (4) running track, (5) outdoor field, (6) outdoor paved area, (7) skating rink/arena, and (8) indoor swimming pool. Positive responses were given a score of 1 and negative responses a score of 0. Scores from all 8 items were summed to create a physical environment score ranging from 0–8. The use of a summary score was used as a previous Canadian HBSC study found that no single specific facility was of particular importance, but that there was a linear relationship between the cumulative number of facilities and student physical activity levels [9].

### School social capital

The HBSC student survey asked students to rate their level of agreement (strongly agree, agree, neither agree nor disagree, disagree, strongly disagree) to the following 4 statements: “*Our school is a nice place to be”, “I feel a lot of trust in my teachers” “Our teachers treat us fairly”*, and *“I feel I belong at this school*”. Responses to individual statements were scored from 0 (strongly disagree) to 4 (strongly agree). Students were also asked “*How do you feel about school at present?*”, and responses were scored from 0 (I don’t like it at all) to 3 (I like it a lot). The responses for all 5 questions were summed to create a 0 to 19 point score, with higher values reflecting higher degrees of school social capital. A school-level social capital score was subsequently derived for each school by calculating the mean of the factor scores from all of the participating students from that school.

The questions *“I feel I belong at this school”*[18] and *“I feel a lot of trust in my teachers”*[19] are based on previous social capital research. The questions *“Our school is a nice place to be”* and *“Our teachers treat us fairly”* have been used to assess school climate in previous HBSC studies [20]. The question *“How do you feel about school at present?”* has been used as an indicator of a student’s perception of school [21]. A factor analysis revealed that these questions represent one factor, which we have called school social capital [22]. The Cronbach’s alpha for this factor derived scale is .81 [22]. Questions had a factor loading ranging from .64 to .83 with the exception of the question *“How do you feel about school at present”,* which had a factor loading of .57.

### Potential confounders

Both student- and school-level confounders were considered. Student-level confounders included socioeconomic status, grade, and gender. Socioeconomic status was determined in the HBSC using the Family Affluence Scale (FAS), which is comprised of four items: vehicle ownership by family, having a bedroom for yourself, family vacations during the past year, and computer ownership. Responses to these items were used to create a 3-point Family Affluence Scale (low, medium, and high) [23]. The FAS has good criterion validity and is less affected by non-response bias then other socioeconomic measures [24]. School-level confounders included urban–rural school location and school size; previous research has shown that urban–rural location is associated with physical activity [25] and school size is associated with student well-being [26]. Based on the population of the municipality where the schools were located, they were classified as being in a rural area (0 – 999 people), small city (1000–29,999 people), medium city (30,000 - 99,999 people), or metropolitan area (≥100,000 people). Principals reported the number of students attending their school, and schools were divided small, medium, and large populations using tertiles.

### Statistical analysis

Analyses were performed in SPSS version 20 [27]. Descriptive statistics including frequencies, means, and standard deviations were conducted. The bivariate relationship between school physical environment and school social capital was determined using a Pearson correlation. Relationships between the exposure variables and the MVPA outcome were examined using multi-level linear regression models that accounted for the clustered and hierarchical nature of the data. The method of estimation was a restricted maximum likelihood procedure. In order to prevent multi-collinearity in the model, school physical environment and school social capital scores were centred [28]. Backwards deletion was used to build a model for the main exposure variables (built environment score and school social capital score) that only included the relevant covariates. The model building started with all candidate covariates. If deletion of the covariate caused less than a 10% change in the effect estimate for either of the main exposure variables, the potential covariate was not included in the model [29]. This process was repeated with all potential covariates. A second model included the variables that were included in model 1 and an interaction term between the school physical environment score and the school social capital score. Next, two stratified analyses were performed. The first stratified analyses examined the association between the school physical environment score and physical activity within low, medium, and high school social capital groups. The second stratified analyses examined the association between the school social capital score and physical activity within low, moderate, and high built environment groups. Low, moderate, and high tertiles were created for these stratified analyses. Finally, the combined influence of the school physical environment and school social capital on MVPA was performed by creating 4 groups: low physical environment/low social capital, low physical environment/high social capital, high physical environment/low school social capital, and high physical environment/high social capital. For these groups, low and high were defined based on the median scores. To determine if there was a difference between these 4 groups, a one way ANOVA with a Bonferroni post-hoc was conducted.

## Results

The distribution of the student participants according to demographic characteristics are shown in Table 1. The sample was fairly evenly split across the two genders and five grade groups. On average, students reported participating in 4.4 ± 3.5 hours/week of MVPA at school. Thirty percent of schools had at least four of the eight physical environment features that were assessed (Table 2). The average school social capital score was 12.6 ± 1.3, with a minimum score of 7.5 and a maximum score of 17.0 (Table 2). The school built environment and school social capital scores were negatively correlated with each other (r = −.19, *p* < .001).

**Table 1:** Distribution of the student sample according to individual-level variables (N = 18,875)

	**N**	**%**
**Gender**		
Male	9051	48
Female	9824	52
**Grade**		
≤6	3697	20
7	3576	19
8	3854	20
9	3960	21
≥10	3788	20
**Family affluence**		
Low	494	3
Moderate	5889	36
High	12492	61
**Physical activity at school**		
Low (<2 hours/week)	5329	28
Medium (2–5 hours/week)	6076	32
High (≥6 hours/week)	7470	40

**Table 2:** Distribution of the schools according to school-level variables (N = 331)

	**N**	**%**
School physical environment features present		
Gymnasium	321	96
Other large room suitable for physical activity	202	60
Fitness room for aerobic or strength training	146	44
Running track	99	30
Outdoor field	280	84
Outdoor paved area	206	62
Skating rink/arena	39	12
Indoor swimming pool	19	6
School physical environment score		
0 (low)	2	<1
1	21	6
2	32	10
3	87	26
4	88	27
5	54	16
6	32	10
7	11	3
8 (high)	4	1
School social capital score		
Low (≤11.93)	6388	34
Moderate (11.94 – 13.11)	6520	35
High (≥13.12)	5967	31

The association between the school physical environment, school social capital, and student physical activity levels is shown in Table 3. As shown in model 1, after controlling for relevant covariates, each one unit increase in the physical environment score was associated with a .040 hour/week increase in MVPA performed at school (*p* = .005) and each one unit increase in the school social capital score was associated with a .074 hour/week increase in MVPA performed at school (*p* = .001). An interaction term between the school physical environment and school social capital scores was added to model 2 (Table 3). There was a minimal change in the parameter estimates for the physical environment and social capital score versus those observed in model 1. The interaction term did not contribute significantly to the model (*p* = 0.192). This suggested that the association between the school physical environment and student MVPA was not moderated by the school social capital.

**Table 3:** Multi-level regression analyses of the association between the school physical environment, school social capital, and moderate-to-vigorous physical activity at school

	**Model 1**			**Model 2**		
	**β**	**SE**	**Pvalue**	**β**	**SE**	**P value**
Physical environment score	.040	.015	.005	.044	.015	.003
Social capital score	.074	.021	.001	.077	.022	001
School size						
Large (referent)						
Medium	.190	.063	.003	.190	.063	.002
Small	.344	.071	<.001	.346	.071	<.001
Grade	-.018	.020	.395	-.020	.021	.356
Physical environment score X	N/A	N/A	N/A	.015	.011	.192
Social capital score						

Note: β coefficient represent the change in hours per week of moderate-to-vigorous physical activity at school per each one unit change in the school physical environment score, one unit change in the school social capital scores, one grade level change, or the schools with a medium or small population relative to the schools with a large population.

**Figure 1:**
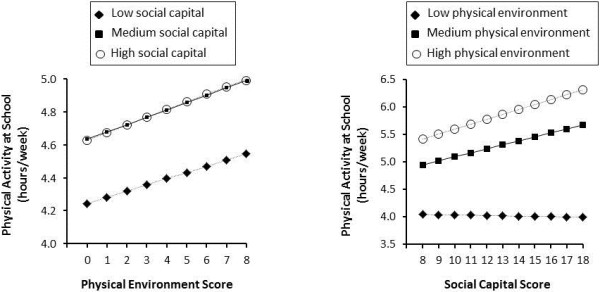
**The association between the school physical environment score with student MVPA levels at school within schools with low, medium, and high school social capital scores (*left panel*) and the association between the school social capital score with student MVPA levels within school with low, moderate, and high physical environment scores (*right panel*).** Data are plotted to represent a grade 8 student from a medium sized school.

To further investigate interaction, a stratified analysis was performed in which the association between the school physical environment and MVPA was examined separately within schools with low, medium, and high social capital scores. As shown in the left panel of Figure 1, there was a positive association between the school physical environment score and student MVPA levels for schools with low (β *= .*038, *p =* .085), medium (β *= .*045, *p* = .086), and high (β *=* .047, *p =* .121) school social capital scores; although these did not reach statistical significance. A second stratified analysis was performed in which the association between school social capital and MVPA was examined within schools with low, moderate, and high physical environment scores. As shown in the right panel of Figure 1, there was no association (β = −.005*, p* = .924) between the school social capital score with student MVPA levels for schools with low built environment scores. Conversely, there was a positive association between the school social capital score with student MVPA levels for schools with moderate (β = .073*, p* = .031) and high (β = .090*, p* = .007) built environment scores.

**Figure 2:**
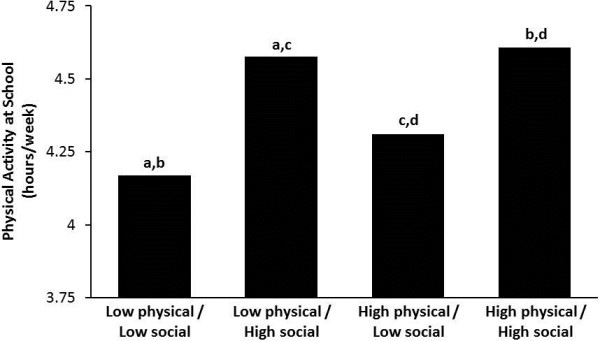
**MVPA levels for students based on low and high physical environment and social capital scores.** The letters on the bars indicate significant group differences; groups with the same letter are significantly (*p* < 0.05) different from each other. For example, the letter “a” indicates that the low physical environment/low social capital group is significantly different from the low physical environment/high social capital group.

Figure 2 shows the average MVPA for schools with low physical environment/low social capital scores (4.17 hrs/wk), low physical environment/high social capital scores (4.57 hrs/wk), high physical environment/low social capital scores (4.34 hrs/wk), and high physical environment/high social capital scores (4.58 hrs/wk). MVPA was significantly (*p* < .05) different across the four groups with two exceptions: the low physical environment/high social capital group was not different from the high physical environment/high social capital group, and the low physical environment/low school social capital group was not different from high physical environment/low social capital group.

## Discussion

The school physical environment and school social capital were both positively associated with students MVPA levels at school. The relationship between the school physical environment and MVPA was not moderated by school social capital.

Our observation that students’ MVPA at school was associated with the physical environment at their school is consistent with previous research which has found a positive association with the amount of school facilities and student MVPA [8,9,30]. The findings of our regression analyses suggests that the difference in physical environments equate to about 20 minutes/week of MVPA for students attending schools with the lowest number of physical environment features by comparison to students attending schools with the highest number of physical environment features. Twenty minutes/week represents a modest amount of MVPA for an individual student; however, if you apply this volume of activity to all students attending schools with few physical environment features the amount is quite meaningful. In fact, about 16% of schools had 2 or fewer of the 8 built environment features, implying that a high prevalence of Canadian students are exposed to poor physical school environments.

The results from this study support a growing body of literature that have linked social capital and physical activity with children and adolescents [11,12,14]. Our study extends these earlier finding as we examined a large and representative group of adolescents and studied the social capital of schools. The findings of our regression analyses suggests that the average weekly volume of MVPA performed by students attending schools with the highest school social capital score is about 40 minutes/week higher than for students attending schools with the lowest school social capital score. Forty minutes/week represents a large amount of MVPA when applied to a large group of students. The findings of our analyses also suggest that school social capital is more strongly associated with MVPA at school than is the school physical environment. This could possibly be explained by the notion that areas with higher school social capital are friendlier and are more willing to let all students play in games rather than excluding students based on sex [31] or age [32]. It is also possible that schools with a high social capital have excellent communication between students and this may increase the awareness of opportunities to be active [11].

To our knowledge, this is the first study to simultaneously consider the influence of the school physical environment and school social capital on MVPA. Interestingly, the school physical environment and school social capital scores were negatively correlated with each other (r = −.19). This negative correlation may reflect the size of the schools as the student population was positively correlated with the number of physical environment features but negatively correlated with social capital. Our results indicate that both the school physical environment and school social capital were independently associated with MVPA while at school. The finding that the association between the school physical environment and MVPA at school was similar in schools with different social capital levels is consistent with previous research on leisure time physical activity [12]. Conversely, the right panel of Figure 1 shows school social capital was related to physical activity in schools with moderate and high built environment scores but not in schools with low built environment scores. This suggests that a high social capital at school may not support physical activity in the absence of a decent physical environment.

Our findings could help direct intervention efforts aimed at increasing MVPA at schools by improving the school environment. Our findings indicate that school social capital is a stronger correlate of student MVPA levels than is the school physical environment. This finding suggests that interventions should focus on ways to increase social capital as compared to enhancing the physical environment. This knowledge is informative for school boards and school administrators as physical environment interventions are likely far more costly than interventions aimed at improving the social capital at schools. Educators can build social capital at their school by creating lessons about events that involve social capital [33]. These types of lessons may inspire youth to make changes in their own school. Another way to build social capital is by using a stepwise process that involves creating awareness amongst students and teachers of the importance of social capital, created opportunities for students and teachers to become engaged in the school environment, and instruct students and teachers on how to make the most of these opportunities [33].

A notable strength of this study was the large and representative sample of Canadian youth. The results of this study may also be generalizable to other northern industrialized countries with similar physical activity levels, school systems, and sociodeomgraphics. A key limitation of this study is its cross-sectional design. With this design type we cannot infer temporality of the observed association. It is possible that active students represent a positive subgroup of the study body that rate the questions on the school social environment higher. Another important limitation is that the MVPA measure was reported by students and this self-reported measure is only modestly correlated with objective measures [34]. This likely lead to non-differential misclassification and observed associations that were biased towards the null.

## Conclusions

In summary, the findings of this study suggest that the association between the school built environment and MVPA is not moderated by school social capital. This study also suggests that school social capital has a stronger influence on school time MVPA than does the school built environment. These results could help inform school-based interventions aimed at increasing student physical activity levels.

## Abbreviations

FAS: Family affluence scale; HBSC: Health behaviour in school-aged children study; MVPA: Moderate-to-vigorous physical activity.

## Competing interests

The authors declare that they have no competing interests.

## Authors’ contributions

BB and IJ came up with the concept and design of the study. Access to and collection of the data were led by IJ and other HBSC investigators. BB performed the statistical analyses and drafted the manuscript with support and feedback from IJ and ST. All authors read and approved the final manuscript.

## Pre-publication history

The pre-publication history for this paper can be accessed here:

http://www.biomedcentral.com/1471-2458/13/1191/prepub
